# Access to praziquantel in Italy: persistent gaps and ongoing responses amid a Europe-wide shortage

**DOI:** 10.1186/s40249-026-01435-8

**Published:** 2026-04-13

**Authors:** Rosalia Marrone, Cristiano Camponi, Silvia Cammarata, Domenico Di Giorgio, Robert Giovanni Nisticò, Christian Napoli

**Affiliations:** 1 National Institute for Migration and Poverty, Rome, Italy; 2 Italian Medicines Agency, Rome, Italy

**Keywords:** Schistosomiasis, Praziquantel, Essential medicine, Drug shortage, Drug importation, Migrant health, Medicine access, Italy, Infectious diseases of poverty, Neglected tropical diseases

## Abstract

In Italy, praziquantel (PZQ) is not registered for human use and can be imported from European countries where it is authorized, according to a specific ministerial decree. Recent Europe-wide shortages, however, have made this route impractical, resulting in the unavailability of this essential medicine. This editorial summarizes historical gaps in supply, the actions undertaken, and the interim solutions implemented to restore PZQ access in Italy. The Italian experience highlights broader challenges related to essential medicines and underscores the need for coordinated national and European strategies to ensure stable and equitable availability.

**Graphical Abstract:**

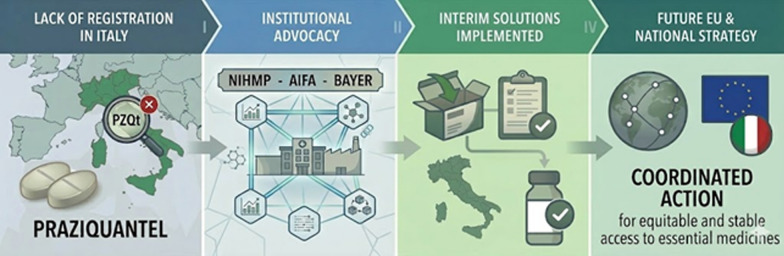

## Praziquantel and the global burden of schistosomiasis

Praziquantel (PZQ) is a broad-spectrum, highly efficacious, and safe anthelmintic agent active against trematode and cestode infections. Developed in the 1970s, it was approved for the treatment of human schistosomiasis in Germany in the early 1980s [1] and has since remained the World Health Organization’s (WHO) recommended drug of choice for all human forms of the disease [2]. Schistosomiasis is considered the most important tropical disease after malaria, affecting millions of people in its intestinal, urinary, and female genital forms. This disease can be particularly severe due to its long-term complications, including bladder cancer and hydronephrosis leading to renal failure, imposing a significant burden on public health systems in terms of healthcare and social costs [3].

## Barriers to access to praziquantel in Italy

Despite the high prevalence of schistosomiasis among migrants in Italy [4] and the existence of national and European recommendations promoting systematic screening [5, 6], access to treatment continues to be limited [7]. Although the World Health Organization (WHO) classifies praziquantel as an essential medicine [8], there has never been a product registered for human use in Italy. Consequently, it can currently only be obtained through a special importation procedure regulated by the Italian Ministry of Health decree of 11 February 1997 [9], which stipulates that importation into national territory is allowed only after the issuance of an authorization by the competent office of maritime, air, border, or internal customs health authorities (USMAF).

## Regulatory framework and procurement procedures

Under this decree, hospitals must enter into agreements with international importers, which has in practice sometimes caused procurement delays and additional costs, affecting timely treatment for highly mobile populations such as migrants.

Established importation flows can help mitigate some of these issues, but delays can still occur, particularly during periods of high demand or shortages.

In this regard, Italy is working to ensure that these “extraordinary” imports can be integrated into already structured flows, thereby simplifying the importation procedure.

The decree establishes that the cost of purchasing medicines cannot be charged to the funds allocated by the State to the regions and autonomous provinces for pharmaceutical assistance, except when the purchase is requested by a hospital facility for use within the hospital setting.

In the latter case, subject to budgetary constraints and any limits established by regional regulations, the hospital may charge the related expense to its own budget, on the same basis as medicines marketed in Italy and other goods necessary for the provision of healthcare services. However, the price of the imported medicine is not negotiated with the Italian Medicine Agency (AIFA) as is the case for medicines reimbursed by the National Health Service (NHS) but is set by the company.

## Supply shortages and the European situation

Biltricide®, manufactured by Bayer, is the only praziquantel formulation approved in Europe, with official national marketing authorization limited to the Netherlands, France, and Germany [10].

However, for several months starting in early 2024, it has not been possible to import PZQ into Italy from other European countries where the medicine is officially registered, due to internal supply shortages [11].

As a result, many Italian hospitals imported PZQ from non-EU countries, particularly from South Asian markets, offering easier access and significantly lower costs, although quality standards may differ from those applied in Europe.

## Institutional response and regulatory initiatives

Given the national significance of the issue and the need to engage with competent authorities, the National Institute for Health, Migration and Poverty (NIHMP), in Rome, has been conducting advocacy since October 2024 targeting AIFA, the regulatory authority responsible for pharmaceuticals in Italy, with the aim of facilitating the registration and commercialization of PZQ.

One of AIFA’s objectives is to promote equitable and timely access to medicines and, since the drug is already authorized for human use in other European countries, the mutual recognition procedure was considered by AIFA to be the most appropriate pathway. In March 2025, Bayer, the marketing authorization holder (MAH) in several European countries, was contacted to assess the company's willingness to support this process. During the same meeting, the company expressed its willingness to support the initiative and to escalate the matter to Bayer AG headquarters.

## Discontinuation of production and emergency supply

Nevertheless, it was not possible to pursue the mutual recognition route as Bayer AG no longer has access to the active pharmaceutical ingredient due to the expiration of its contract with the supplier, and, despite intensive efforts, was unable to identify an alternative source using the same manufacturing process. As a result, Bayer informed the countries where the drug is registered that commercialization has been discontinued and notified the European Medicines Agency (EMA) [12, 13]. This issue was subsequently discussed at the EMA Medicine Shortages SPOC Working Party meetings held on 13 May and 17 June 2025 [12, 13]. Bayer made a final batch available to all countries that reported a need, manufactured in Germany and packaged in the United States.

In December 2025, Italy received a portion of this production sufficient to treat approximately 2400 or 7200 individuals (assuming an average body weight of 70 kg per person administered either as a single dose or over a 3-day treatment) [14].

## Interim national solution for praziquantel importation

Therefore, to allow the timely treatment of patients, following various meetings between AIFA, NIHMP, and MAH, a procedure was established under the Ministry of Health Decree of 11 February 1997. According to this procedure, any Italian hospital or regional authority requiring praziquantel may submit a request for ward stock supplies to the territorially competent USMAF using the designated form pursuant to the Ministry of Health Decree of 11 February 1997, with the advantage of drug stocks already being available in Italy.

Despite having discontinued commercial distribution in some European countries, Bayer’s support during the emergency highlights how public-private collaboration can be pivotal in ensuring timely access to essential medicines.

## Challenges in access to essential medicines in Italy

Approximately 85.2% of medicines on the WHO List of Essential Medicines (EML) were commonly marketed in Italy, however, the coverage is lower for certain classes, particularly anti-parasitic, insecticide, and repellent products [15]. The Italian experience with praziquantel illustrates challenges related to essential medicines that are not registered nationally, highlighting structural limitations and broader market gaps addressed by European initiatives such as the EU Critical Medicines Act.

Since the new pharmaceutical legislation is under approval, it is likely that its national implementation, the relevant authorities will take these aspects into consideration as evidenced by the recent revision of the standard operating procedure on the authorization for the importation in Italy [16].

## Public health implications and future perspectives

Ensuring access to praziquantel, as well as to all essential medicines [15, 17], is crucial for providing effective treatment for emerging and re-emerging diseases, whose incidence is already increasing and is expected to rise further due to migration, international mobility, and climate change. However, current measures remain a short-term solution that addresses an immediate treatment gap but do not resolve the long-term need to guarantee uninterrupted availability of essential medicines. This is particularly important given the risk of autochthonous schistosomiasis in Italy and Europe, as illustrated by well-documented cases in neighbouring Corsica over the past decade [18], underscoring the critical need for continued access to praziquantel.

Since this issue has clearly surpassed national borders, timely, coordinated, and sustained action by Italy and other European countries is essential to protect the right to health and prevent severe public health threats. As highlighted in recent literature [19], inequitable access to praziquantel and other essential medicines for infectious diseases of poverty remains a European challenge with immediate implications for global health, underscoring the urgent need for collective action.

## Data Availability

Not applicable.

## Abbreviations

WHO: World Health Organization
PZQ: Praziquantel
EMA: European Medicines Agency
MAH: Marketing authorization holder
NIHMP: National Institute for Health, Migration and Poverty
AIFA: Italian Medicine Agency
NHS: National Health Service
USMAF: Internal Customs Health Authorities
